# Coxitis in a Mule

**Published:** 1897-12

**Authors:** W. E. A. Wyman

**Affiliations:** Clemson College, South Carolina


### COXITIS IN A MULE.

By W. E. A. Wyman, V.S.,

CLEMSON COLLEGE, SOUTH CAROLINA.

Recently, while investigating for the State an outbreak of hæmoglobinuria among cattle, the writer was shown a mule offering the following symptoms: Severe mixed lameness, supporting-leg lameness being somewhat more marked than swinging-leg lameness. Inspection while standing still revealed decided atrophy of all gluteal muscles of the right hind leg. The great trochanter of the almost vertical femur, which seemed to be in front of the cotyloid cavity, was plainly visible under the skin. The external angle of the ilium of the affected right side was lower than the one of the left side. The leg was abducted, the toe turned out, the point of the hock almost touching the inner face of the left hock. Palpation over the region and in front of the cotyloid cavity seemed very painful, the mule grunting when the parts were handled. Right in front of the coxo-femoral articulation a movable enlargement (movable only toward the cotyloid cavity) was felt. This I took to be the superior extremity of the femur, but was puzzled to a great extent why it could not be moved upward and forward. Rectal examination showed a swelling on the shaft of the ilium, some rough spots were felt along the anterior border of the foramen ovale.

The history of the case is as follows: Nine months ago the mule, four years old, was playing in the lot when it suddenly slipped and fell upon the right side; got up immediately, but was very lame. The animal was then given absolute rest for about six weeks, when the owner found him down in the stable one morning, unable to rise. With some help he got up; his toe was turned out, and something seemed to be loose and working under the skin whenever he stepped upon the lame leg. The owner, thinking something was dislocated, put the mule into deep water and made him swim, hoping that the dislocated part would thus come back into place.

The symptoms and history of the case induced me to make a diagnosis of luxatio iliaca, the result of a primary fissuring of the bones of the cotyloid cavity, and subsequent destruction of the ligamentum teres and dislocation of the femur.

The owner having consented to kill the animal, a post-mortem examination was held, and brought out the following points: The deep gluteus was practically converted into a fibrous capsule which had spread and surrounded all the diseased parts of the shaft of the ilium. The head of the femur was resting in a rough, new, articular cavity right in front of the cotyloid cavity, the roof of that new cavity being formed by an enormous exostosis, which, at the same time, explains why the writer was unable to push the femur upward and forward.

The cotyloid cavity was rough and surrounded by exostoses. The new articular cavity was quite smooth in places. The head of the femur and the whole of the scabrous portion were diseased. After careful study of the bones, the writer has come to the conclusion that his diagnosis of luxatio iliaca, the "result of a primary fissuring of the bones of the cotyloid cavity, etc.," was not correct, but that the ligamentum teres was primarily affected, and subsequently the bony structures.

